# Polymorphisms of vascular endothelial growth factor—2578C/A rs699947 are risk factors for diabetic retinopathy in type-2 diabetes mellitus patients in Bali, Indonesia

**DOI:** 10.37796/2211-8039.1170

**Published:** 2021-06-01

**Authors:** Audrey Rachel Wijaya, I Wayan Surudarma, Desak Made Wihandani, I Wayan Ardyan Sudharta Putra

**Affiliations:** a Faculty of Medicine, Udayana University, Denpasar, Bali, Indonesia; b Department of Biochemistry, Faculty of Medicine, Udayana University, Denpasar, Bali, Indonesia

**Keywords:** Diabetes Mellitus, Diabetic Retinopathy, SNP rs699947, VEGF Polymorphism

## Abstract

**Background:**

Diabetic retinopathy (DR) is one of the complications in diabetes mellitus (DM) which caused by microvascular-damage in the retina due to long termmetabolic changes in diabetes. To date, there has been much research targeted on the determinant of genetic identification in DR patients. In DR, Vascular Endothelial Growth Factor (VEGF) gene is accountable for breaking down the blood-retinal barrier and implicated in the role of neovascularization. It is thought that the polymorphism of VEGF -2578C/A (rs699947) contributed to the development of diabetic retinopathy in type 2 DM.

**Aim:**

To determine whether the polymorphisms of VEGF-2578C/A are the risk factors for DR in type 2 DM patients in Bali, Indonesia.

**Methods:**

This study is a case-control model comparing 33 cases DR patients in type-2 diabetes mellitus and 35 cases of non-DR as controls in Balinese ethnic. Polymorphisms of VEGF-2578C/A were examined by PCR analysis and DNA sequencing on rs699947 to identify any variation in A/C/T allele distribution. Chi-square test was used to analyze the data and determine the relation of polymorphism and DR.

**Results:**

This research showed the genetic variation existence in VEGF-2578C/A polymorphism significantly (p = 0,000) with C allele was higher in the DR group, in contrast, A and T allele were greater in the non-DR group compared to DR group. The result showed that C allele in VEGF-2578 contributed as a risk factor (OR = 13.05; 95% CI = 2.69–63.18; *p* = 0.001) for DR in type-2 DM (T2DM) patients in Bali, Indonesia.

**Conclusion:**

Polymorphism of VEGF-2578C/A (rs699947) allele distribution can be concluded as a risk factor of DR within T2DM patients in Bali, Indonesia. This study may also be used to expand the knowledge in managing DR patients at an earlier stage to avoid further complications.

## 1. Introduction

Diabetes mellitus incidence specifically type-2 diabetes mellitus (T2DM) continuously increasing in both developed and developing countries including Indonesia. In 2019, there were an estimated 463 million cases of T2DM[1] and WHO (2015) estimated that there will be an increase as many as 21.3 million cases by 2030 [2].

T2DM is a multifactorial condition accompanied by hyperglycemia that potentially caused macrovascular and microvascular complications. One of the microvascular complications that may occur is diabetic retinopathy [3,4]. Diabetic retinopathy (DR) is the major contributor to visual disturbances in patients who are still of productive age [5]. The prevalence of DR in patients with diabetes is 22–37%. There is microvascular impairment in the retina due to persistent metabolic changes exposure in diabetes. If the condition is allowed to continue, it can lead to blindness [6,7]. In 2015, DR was estimated to be the seventh leading cause of blindness worldwide [8]. Patients with controlled levels of blood sugar can develop DR complications even in their early phases of diabetes. However, some other patients with years of diabetes do not develop DR complications. These demonstrated the fact that the incidence of DR is not only caused by hyperglycemia. Several studies have shown a role for genetics in DR [7,9,10].

VEGF is an angiogenesis and growth factor that is closely related to vascular permeability in endothelial cells [11]. Activation of VEGF is associated with induced-microvascular changes in hypoxaemic conditions in DM patients or hyperglycemic conditions [12,13]. VEGF activation causes significant damage to the blood-retinal barrier and can also induce neovascularization in proliferative diabetic retinopathy [14,15]. Several studies suggest that the presence of a single nucleotide polymorphism (SNP) −2578 in the VEGF promoter region can affect the development of diabetic retinopathy. However, some results of studies differ from many countries [16–20]. These varying results can be caused by ethnic differences factors [21].

Polymorphism of the VEGF -2578C/A gene is one of the polymorphisms that occur in the promoter region of the 5′ untranslated region (UTR) [22,23]. The promoter is located in the upstream area of the regulated gene and is the site of binding to activators or transcription factors. Several sites that bind to transcription factors are found in the 5′ UTR and polymorphisms in this region cause differences in VEGF expression [23]. There are 3 references of allele variations in rs699947, namely A/C/T with the A allele as the ancestral allele [24]. This study aims to determine the polymorphism relationship of VEGF-2578C/A (rs699947) as a risk factor for DR in type 2 DM patients in Bali, Indonesia.

## 2. Methods

### 2.1. Study subject

This study is a case-control study which already approved by institutional ethics committee (289/UN14.2.2.VII.14/LP/2020). It aims to determine the polymorphisms that were found are risk factors for diabetic retinopathy in T2DM patients in Bali. Sample size planning was calculated based on reference with a total of 68 samples (33 T2DM patients with DR and 35 T2DM patients without DR) were used in this study using stored biological material from previous studies [7] at the Department of Biochemistry, Faculty of Medicine, Udayana University in the form of DNA isolates of type 2 diabetes mellitus patients in Bali.

### 2.2. Polymorphism detection

**Fig. 1:**
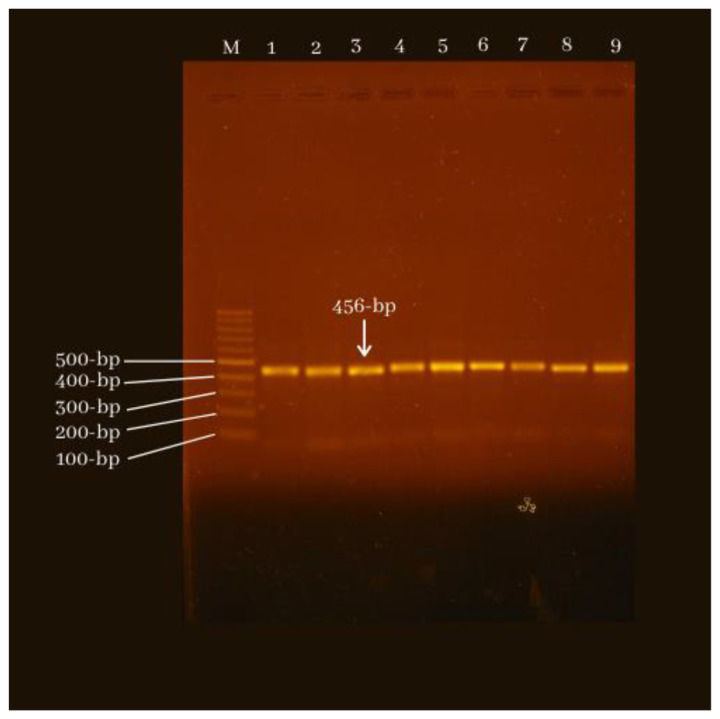
PCR result visualized in gel electrophoresis.

Polymorphisms were detected by Polymerase Chain Reaction (PCR) analysis followed by DNA sequencing. The VEGF gene promoter fragment with polymorphism −2578C/A was amplified by PCR using forward primers: 5′-GGCCTTAGG ACACCATACC-3′ and reverse primers: 5′-CACAGCTTCTCCCCTATCC-3′, amplifying fragments of 456 bp. PCR was performed using the GoTaq® Green Master Mix PCR Kit from Promega. Amplification was carried out in 40 cycles each consisting of predenaturation of 95°C for 10 minutes, denaturation of 94°C for 35 seconds, annealing 58°C for 40 seconds, 72°C for 45 seconds, and extension 72°C for 10 minutes. The PCR product results are then sequenced (Fig. 1). Polymorphisms were analyzed based on electropherograms confirmed by BLAST with reference sequences in the gene bank [24].

### 2.3. Statistical analysis

The data obtained were entered in a master table for analysis using the computer program SPSS 25.0 with clinical data summarized with the mean ± standard deviation between cases and controls and the normality of the variables was tested by independent t- and Mann-Whitney U tests. Chi-square test was conducted to prove the association between diabetic retinopathy variables and polymorphism of VEGF -2578 C/A with a significance of p < 0.005.

## 3. Results

Because this study used the samples of stored biological material, the data of basic characteristics of the sample have been obtained and have been analyzed, The univariate description can be seen in Table 1.

**Fig. 2:**
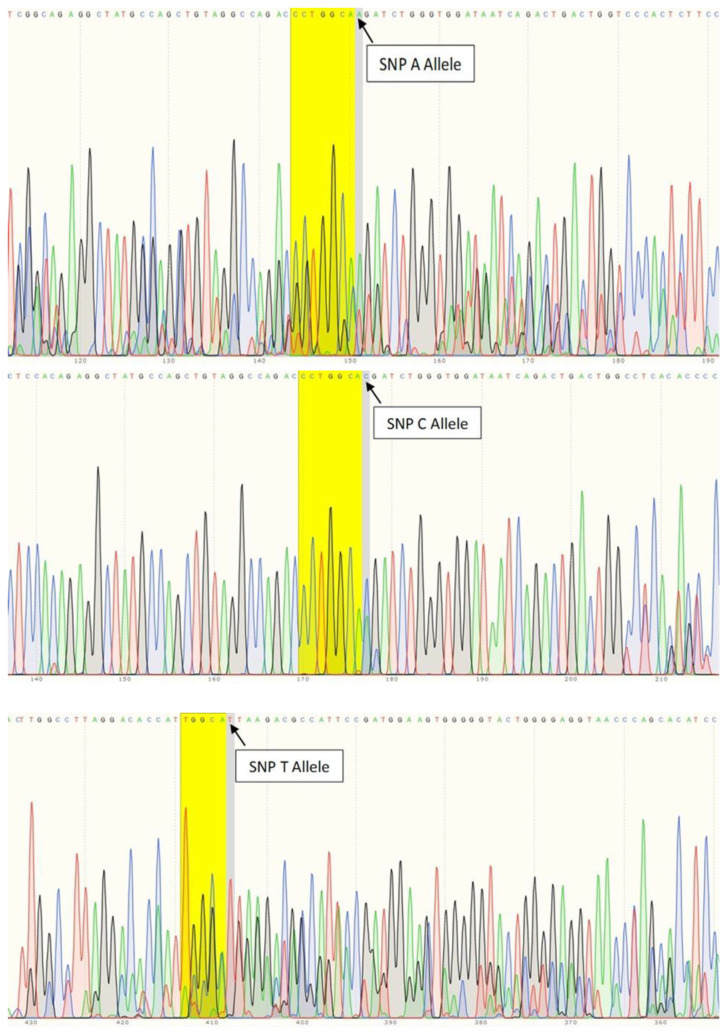
Electropherogram showing single nucleotide variation in rs69994. (a) Electropherogram of SNP A allele. (b) Electropherogram of C allele. (c) Electropherogram of T allele.

According to Table 2, in the DR (+) case group with a total of 33 samples, we found that there were 31 samples of C allele (93.9%), 1 A allele (3%), and 1 T allele (3%). Whereas in the DR (−) control group with a total of 35 samples, there were 19 samples of C allele variation (54.3%), 6 samples of the A allele (17.1%), and 10 samples of T allele (28.6%). These polymorphisms were determined by DNA sequencing as shown in Fig. 2.

We found that there was a significant association of polymorphism of VEGF-2578C/A (rs699947) in the DR and non-DR groups with high allele polymorphism variation in the DR group, while the A and T allele were higher in the non-DR group in T2DM patients in Bali (OR = 13.05; 95% CI = 2.69–63.18; p = 0.001) as shown in Table 3.

## 4. Discussion

The allele variations in the rs699947 region can cause differences in VEGF gene expression. It refers to the clinical differences between one patient and another. Based on several studies that have been conducted, there were significant and insignificant variations in the association between SNP VEGF at nucleotide position −2578 (rs699947) with variations in C and A alleles which are associated as risk factors for DR in various populations [21,25,26]. A meta-analysis of eight studies in 2,402 patients in Asia and Europe, as well as 500 patients study in China, declared that the polymorphism rs699947 VEGF-2578C/A significantly associates with DR in T2DM individuals [18,19]. According to a meta-analysis study with 1,702 Caucasian and Asian patients, the correlation of rs699947 (−2578C/A) and DR polymorphisms was significant for Asians but not for Caucasians [20]. However, a meta-analysis of 2,208 Asian and Caucasian patients, 126 Egyptian patients, and a combined study of 1,040 Chinese ethnicity patients, found no significant association of rs699947 (−2578C/A) polymorphism with DR in T2DM patients [16,17,27,28].

In this study, a major difference in allele frequency was discovered (p = 0.000) on SNP VEGF-2578 (rs699947) where the C allele was the most common allele variation found in both the DR and non-DR groups. This is similar to several other studies in various countries regarding the relationship of polymorphism of VEGF rs699947 with DR on T2DM [21,25,29,30]. Besides, this study also found variations in the A and T alleles in the DR and non-DR groups respectively. In this case, there have not been any other studies that discuss the relationship of the T allele in the polymorphism of VEGF-2578 (rs699947) with DR.

Based on the analysis of VEGF-2578 rs699947 polymorphism association with DR within T2DM patients in Bali, it was found that there was a significant relationship with a higher percentage of C allele in the DR group compared to non-DR. Besides, A and T alleles were found higher in the non-DR group compared to DR. It showed that the C allele plays a role as a risk factor for DR in T2DM patients in Bali (OR = 13.05; 95% CI = 2.69–63.18; p = 0.001). This association is in line with the C allele which facilitates the binding site for hypoxia-inducible factor 1 alpha (H1F1α). On the other hand, A allele reduces the number of those binding sites [31,32]. H1F1 takes place as a major component in the activation of VEGF gene expression induced by hypoxia, as it binds to the hypoxia responsive element (HRE) [33–35]. Excessive VEGF expression in a hypoxic state results in proliferation and migration of retinal endothelial cell and retinal vascular leakage which lead to neovascularization [15,36,37].

Several other studies that have been carried out in various populations of countries to discover the relation between the VEGF-2578 gene (rs699947) and DR had various results. Chun et al., (2010) discovered a considerably greater percentage of the C allele within the non-DR group, while the percentage of A allele is more in the DR group, thus it indicates a protective nature of the C allele at rs699947 in the incidence of DR in the Korean population [30]. The same thing was also found significant in the study of Nakamura et al., (2009) in Japan with the percentage of C allele more in the non-DR group and the A allele in the DR group. They estimated that the A allele on SNP VEGF-2578 was associated with proliferative DR by regulating increased mRNA levels of the VEGF gene which subsequently increased intraocular VEGF synthesis [21].

The difference in the results of these studies can be caused by ethnic differences between the study samples [20,38,39]. In general, the frequency of allele susceptibility varies in each population. Thus, the different results could be due to differences in allele frequencies among the study population [40,41].

## 5. Conclusion

We found that there was a significant association in polymorphisms of VEGF-2578C/A rs699947 as risk factors of DR. Three alleles were found, *i.e*. A, C, and T allele. C allele was the risk factor in developing DR, while A and T allele were found to be the protective factors for the incidence of DR within T2DM patients in Bali, Indonesia.

## Figures and Tables

**Table 1:** Characteristic between Case and Control Group.

Characteristic	DR N = 33	Non-DR N = 35	p
Sex			
Male (n)	21	19	0.434
Female (n)	12	16	
Age (years)	50.15 ± 7.02	54.71 ± 9.86	0.031
Duration of DM (years)	7.42 ± 3.61	6.1 ± 5.36	0.036
Family history of DM			
Yes (n)	24	21	0.268
No (n)	9	14	
BMI (kg/m2)	24.58 ± 3.04	25.2 ± 4.27	0.495
Waist Circumference (cm)	93.03 ± 7.43	90.45 ± 11.35	0.184
HbA1c (%)	8.66 ± 2.03	8.72 ± 2.96	0.488

Significance at *p* < 0.005.

**Table 2:** Allele Distribution of VEGF -2578C/A Polymorphism Gene.

Variable	Allele A	Allele C	Allele T	p
DR (+)	1 (3.0%)	31 (93.9%)	1 (3.0%)	0.000
DR (−)	6 (17.1%)	19 (54.3%)	10 (28.6%)	

Significance at *p* < 0.005.

**Table 3:** VEGF −2578C/A Polymorphism Gene for Case and Control as Risk Factor for DR.

Allele	DR (+) n = 33	DR (−) n = 35	Odds ratio (95% CI)	p value
C	31 (93.9%)	19 (54.3%)	13.053 (2.69–63.18)	0.001
A + T	2 (6.1%)	16 (45.7%)		

Significance at *p* < 0.005.
